# Grade III of Blunt Thoracic Aortic Injury and Duodenal Perforation due to Multiple Trauma; Which Is Priority? A Case Report

**DOI:** 10.1002/ccr3.70025

**Published:** 2025-01-09

**Authors:** Nasser Malekpour Alamdari, Iman Ansari, Hamed Askarpour, Maryam Abbasi

**Affiliations:** ^1^ Critical Care Quality Improvement Research Center, Shahid Modarres Hospital Shahid Beheshti University of Medical Sciences Tehran Iran; ^2^ Department of General Surgery, Shahid Modarres Hospital Shahid Beheshti University of Medical Sciences Tehran Iran; ^3^ Department of Heart Surgery, Shahid Modarres Hospital Shahid Beheshti University of Medical Sciences Tehran Iran

**Keywords:** blunt thoracic aortic injury, duodenal perforation, multiple trauma, thoracic endovascular aortic repair

## Abstract

Perforation of the duodenum after trauma has a low incidence and its coincidence with BTAI is very rare. The clinical condition of the patient is very important for deciding the treatment priority. In this patient, considering the stable condition and degree of aorta injury, we chose TEVAR before laparotomy.

## 1 Introduction

The most common cause of death in young people is trauma [1]. Perforation of the duodenum due to trauma usually occurs after high energy abdominal trauma and it has a very low incidence especially in adults, although its mortality is about 30% [2]. If the intensity of the trauma is high, the possibility of damage to other parts of the body, including the chest, increases. Although blunt thoracic aortic injury (BTAI) occurs only in 0.3% of severe traumas, BTAI is the most common cause of death after head injury following trauma [1, 3]. Only 20% of patients who experience BTAI reach the hospital alive, and about half of survivors die within the first 24 h [4]. BTAI is divided into four categories by the Society of Vascular Surgery (SVS). Grade I, intimal tear; Grade II, intramural hematoma; Grade III, pseudoaneurysm; and Grade IV, free rupture [5]. Grade I and II are usually treated conservatively. But higher grades requires surgery. Unfortunately, despite progress in treatment, many cases of grade IV die [1]. The surgical approach in BTAI includes open surgery and thoracic endovascular aortic repair (TEVAR). During the last decade, TEVAR has replaced open surgery as an effective first‐line treatment for BTAI [6]. Despite the preference of vascular surgeons for TEVAR, the timing of TEVAR for the treatment of BTAI has been controversial. In some studies, it has been suggested that it is better to perform TEVAR electively, and in other studies, there was no significant difference between the time of performing it [7, 8]. In addition, the results of a 20‐year review have shown that a conservative approach to BTAI has been associated with increased mortality [9]. Deciding on the time of treatment becomes more important when there is a concomitant injury that requires immediate treatment. In this study, we report a rare case of BTAI with perforation of the duodenum, and according to the patient's clinical conditions, we decided to prioritize the repair of the aorta instead of the duodenum, and the TEVAR approach instead of open surgery to repair the aorta.

## 2 Case History/Examination

A 26‐year‐old male patient with no history of underlying disease or surgery was sent to the emergency room by ambulance following multiple trauma after a car accident. The time interval between the accident and entering the emergency room was about 30 min. At the time of visit, the patient was alert and anxious. He complained of pain in his limbs, pelvis and abdomen. He did not feel nausea. Vital sign at presentation was pulse rate: 105; blood pressure: 95/60; respiratory rate: 24; temperature: 36.7°C; saturated O_2_: 95%; and Glascow Coma scale: 15. The patient was immediately given basic resuscitation. The airway was normal. The patient was breathing spontaneously. The breathing rate was high, but there was no respiratory distress. There was no evidence of damage to the skull or mouth. The trachea was in the middle line. Chest movements were symmetrical. No sound reduction was heard during lung auscultation. He did not have neck or chest emphysema. There was no evidence of neck hematoma. Jugular venous pressure was normal. There was no evidence of active bleeding in any part of the body. On more detailed examination, there was evidence of a small laceration on the right eyebrow. Heart auscultation had no pathological findings. The abdomen was smooth, there was no distention. It had no guarding or rebounds. There was generalized abdominal tenderness, especially in the epigastrium. There was ecchymosis in the perineum and scrotum. Movement of the patient's pelvis was painful. The limbs were warm and the pulse was symmetric and normal. The sensation and movement of the limbs was acceptable. He had tenderness in the heel of his right foot.

## 3 Methods

**FIGURE 1:**
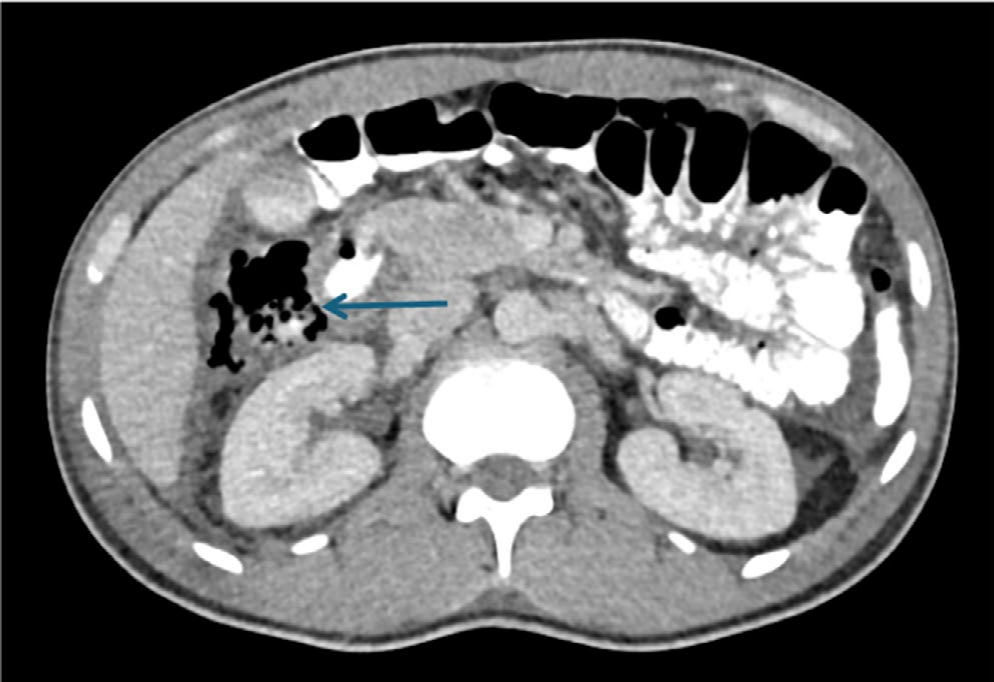
Evidence of pneumoretroperitoneum below the liver and around the duodenum in computed tomography imaging (arrow).

Focused assessment with sonography in trauma (FAST) was performed and there was evidence of minimal to mild perisplenic, perihepatic and Morrison's fluid. After establishing stable conditions, the patient underwent imaging workup. In the graphs, there was evidence of a fracture of the pubic ramus on the right side and the right calcaneus bone. There was no pathological point in the computed tomography (CT) without contrast of the brain and neck. In chest CT with contrast, there was evidence of outpouching of the anterior inner wall of the descending aorta at the level of ligamentum arteriosum measuring 19 × 11 mm. In the abdominal CT with contrast, there was evidence of pneumoretroperitoneum around the second part of the duodenum and subhepatic area (Figure 1). In addition, partial laceration with hematoma was evident in the spleen and mild perisplenic fluid. There was mild interloop fluid in the abdomen and pelvis. Also, the fracture of the pubic ramus on the right side and the surrounding soft tissue hematoma were evident.

**FIGURE 2:**
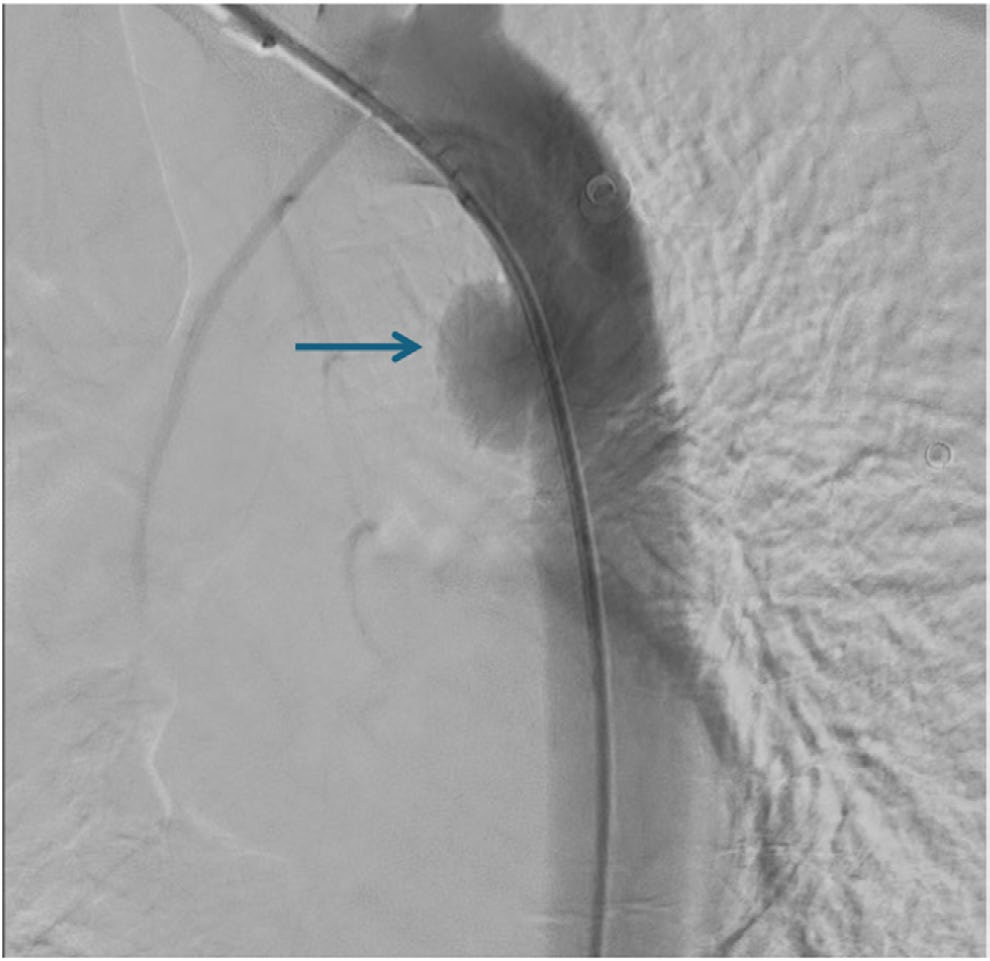
Evidence of grade III of blunt thoracic aortic injury (arrow) in digital angiography.

**FIGURE 3:**
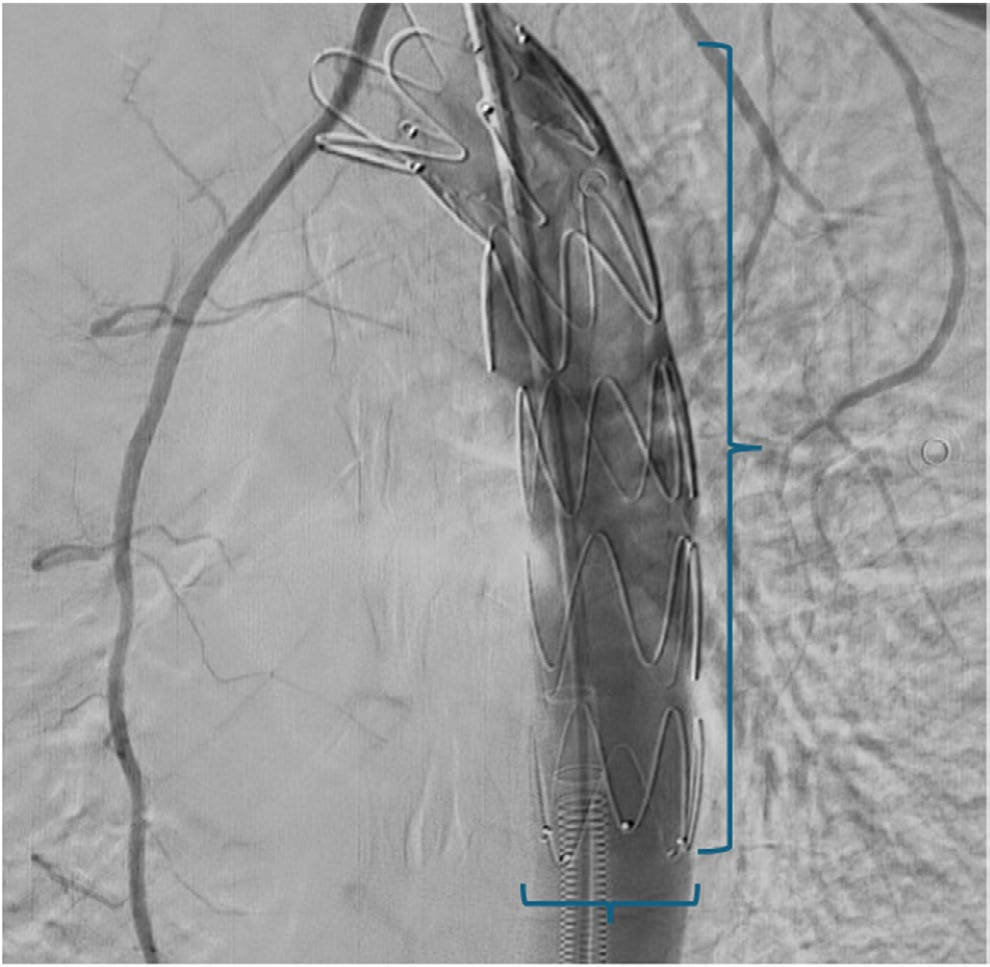
Successful repair of the descending aorta using TEVAR method and stent implantation (brackets).

Due to the absence of symptoms of peritonitis, we decided to repair the aorta at this stage. The patient was a candidate for TEVAR. Digital angiography of the descending thoracic aorta and aortic arch was performed under general anesthesia, and the diagnosis of BTAI grade III of the descending aorta after the left subclavian location was confirmed (Figure 2). After inserting the F6 pigtail, in the right common femoral artery to ascending aorta, the hydrophilic guide wire was advanced to the aortic arch, and the wire was replaced with Lunderquist Wire. The Zenith Alpha stent was released in the right place under X‐ray positioning. Control arterial angiography was normal. Next, the femoral artery was repaired (Figure 3).

After performing TEVAR, the patient was transferred to the intensive care unit and was monitored for 12 h. During this interval, the clinical condition of the patient was stable. Later, the patient was a candidate for exploratory laparotomy. On examination of the abdominal cavity, there was evidence of brief fluid and partial splenic hematoma. Stomach and liver had no pathological findings. From the ligament of Trietz to the ileocecal valve and then to the rectum, there was no significant point. There was a hematoma and minimal bile discharge around the duodenum and subhepatic area. In further investigation, there was a 1 cm perforation on the lateral wall of the second part of the duodenum, which was initially repaired by simple suture. The omental flap was placed on the repair site. A gastrojejunostomy was performed to protect the repair. After making sure that there was no damage in other parts of the abdomen, the surgery was ended. Five days after the surgery and after ensuring recovery, the patient was transferred to the orthopedic service to continue the treatment. In the follow‐up 3 months after the surgery, the patient did not complain.

## 4 Discussion

BTAI is considered a surgical emergency and although it is a rare event following trauma, is associated with high mortality [4]. The results of the investigations have shown that the most likely injury occurs in the proximal part of the descending aorta, followed by the ascending aorta, the aortic arch, and the middle and distal parts of the descending aorta. In about 15% of cases, the thoracic aorta may be injured in several places [10]. The main mechanism of injury is rapid deceleration and mostly follows motor vehicle accidents. There are generally three approaches to treating BTAI: conservative treatment, TEVAR, and open surgery. High bleeding rate, neurologic events, higher mortality, and longer recovery in open surgery have led to more popularity of TEVAR. But still, in cases where the anatomical conditions are not suitable for TEVAR and in some emergency cases especially in combat injuries, open surgery is indicated [1, 11]. In this method, the chest is usually opened by left thoracotomy and after appropriate vascular control, the aortic graft is implanted. More complications of this method have limited its use in the treatment of BTAI. A 9‐year review by Branco et al. has shown that the use of endovascular methods for aortic repair has expanded, especially in severe blunt traumatic injuries [12].

The patient of the present study had referred to our center due to multiple trauma after a car accident. In the investigations, grade III of BTAI along with pneumoretroperitoneum the possibility of duodenal perforation were considered. According to the results of the studies, considering that BTAI usually occurs after high energy trauma, another serious injury may also be involved and it challenges the decision for treatment priority [4]. Considering the grade of BTAI, we decided to perform surgery instead of conservative treatment. Also, according to the stable hemodynamics of the patient and the appropriate anatomical condition of the aorta, our option for the treatment of BTAI was TEVAR. In addition, due to the absence of symptoms of peritonitis we decided to postpone the abdominal surgery until after the aortic repair. Both surgeries were successfully performed for the patient, and the patient did not experience any complications after 3 months. In a study conducted on 275 patients with BTAI requiring surgery, the mortality rate in the group that underwent TEVAR was lower than open surgery [6]. Minici et al.'s follow‐up with an average of 80 months in 38 patients who underwent TEVAR for the treatment of BTAI, no serious postoperative complications were observed and 0% mortality was reported, which shows the importance of TEVAR in the treatment of BTAI and is similar to the results of the present study [13]. The results of Al‐Thani et al.'s 20‐year experience showed that mortality in a group of BTAI patients who underwent open surgery or conservative treatment was significantly higher than the group who underwent TEVAR [9]. Therefore, it seems that conservative treatment in the patient of the present study may be associated with increased mortality risk. On the other hand, in the absence of peritonitis symptoms, delayed abdominal surgery in patients with concomitant blunt abdominal injury may have other benefits. The results of Hsu et al.'s study showed that some cases of abdominal trauma with BTAI may be managed conservatively, but may require abdominal surgery after TEVAR [14]. Lu et al. reported that the incidence of intra‐abdominal hemorrhage after TEVAR is higher in patients who have BTAI and blunt abdominal injury at the same time, and also in these patients, TEVAR increases the risk of delayed abdominal surgery [15]. Therefore, it will be possible to control intra‐abdominal bleeding after TEVAR with delayed abdominal surgery, and on the other hand, the need for repeat laparotomy will be reduced. Despite the mentioned findings, it is not possible to draw a definite conclusion to determine the best treatment method from case report studies, which is one of the limitations of the present study, so more studies with sufficient sample size are suggested in this field.

## 5 Conclusion

Perforation of the duodenum after trauma has a low incidence and its coincidence with BTAI is very rare. The clinical condition of the patient is very important for deciding the treatment priority. In the present study, we experienced a successful delayed laparotomy after emergent TEVAR. But the treatment priority may be different in other conditions. Therefore, a definite conclusion for the priority of treatment requires more studies.

## Author Contributions

**Nasser Malekpour Alamdari:** conceptualization, investigation, supervision, writing – original draft, writing – review and editing. **Iman Ansari:** data curation, investigation, methodology, project administration, validation, writing – original draft, writing – review and editing. **Hamed Askarpour:** conceptualization, investigation, visualization, writing – review and editing. **Maryam Abbasi:** conceptualization, formal analysis, project administration, resources, writing – original draft, writing – review and editing.

## Ethics Statement

The authors tried to keep the information of the patient confidential and the results were published anonymously.

## Consent

Signed consent form was also obtained from the patient.

## Conflicts of Interest

The authors declare no conflicts of interest.

## Data Availability

The data that support the findings of this study are available on request from the corresponding author. The data are not publicly available due to privacy or ethical restrictions.
